# The Field of Science

**Published:** 1893-11-25

**Authors:** 


### The Field of Science.

The cremation furnaces of Père Lachaise have been utilized during the last four years for the disposal of the anatomic remains of the Parisian hospitals and dissecting rooms. During this period no less than 111,852 incinerations of this kind have been executed at the cemetery as against a much lesser proportion of the order for which the furnaces were originally designed.

A curious instance of the first vaccination case performed in Russia comes to us from America. The Empress Catharine II. submitted to being the pioneer of the practise of inoculation in her own domains, the preparations for the novel function being elaborately worked out. The lymph was supplied by a boy of seven years of age named Markof. The child was subsequently ennobled under the fancy name of Ospiennyi (Ospa means small-pox), and he was brought up under the Empress' own tutelage. At the present day the boy's descendants occupy a prominent position among the Russian aristocracy.

Although the bacterial contents of waters derived from such different sources as lakes, rivers, springs, and wells have had the eye of the analyst turned in all vigilance on them, yet, as a contemporary remarks, the microbial condition of sea water has been less efficiently ascertained, or rather, has attracted less attention. Giaxa, who is one of the earliest researchers in this direction, records that after careful analysis, he failed to find more than ten organisms in one cubic centimetre, at a mile and a half off the coast of Naples. Sea water is singularly clear of bacteria, but the poverty, so to speak, of the water is not borne out in the sea mud, in which as many as 245,000 microbes have been discovered in one cubic centimetre of slime, at a depth of 164 feet. Bacteria was further found in considerable numbers on the edge of the great continental platform which skirts the Gulf Stream; here the two prevailing kinds being the same as those discovered by the Massachusetts Coast, the observations along which sea-line we owe to Mr. Russell, who has lately extended his investigations in this direction.

Dr. Haffkine is pursuing in an indomitable manner his anti-cholera precautions in India. At present he is following in the wake of the Hardwak Pilgrims, and is steadily going through the North-West Provinces. Much sympathy has been expressed with his work from many quarters, and one very practical form of this has found expression in the instance of the Governor of Naini Tal, who, we learn, has provided that a Deputy Sanitary Commissioner be placed at Dr. Haffkine's disposal, as also a Tashuldar, to interpret to the natives the motive of the Doctor's precautionary measures. So far, the natives have proved amenable to reason, and ready to submit to the harmless operation, and many thousands have consented to become thus inoculated. Dr. Haffkine has organised a system of correspondents at each station, who undertake the preliminary measures, ascertaining the number of inhabitants willing to undergo operation, a proper record of which number is duly kept, as also of the immediate results contingent on the preventive measures.

The demand for indiarubber is fast assuming such immense proportions that efforts are being made to extend the cultivation of the trees in order to keep pace with the demand. The trees in Trinidad, we hear, are now beginning to yield considerable quantities. Dr. Ernst, from the Orinico, makes a timely suggestion, and one which should be duly attended to. This is that collectors who are working on any particular grove should be compelled before beginning an operation which ends in the total destruction of the trees to prepare a fresh plantation to act as reserve property, otherwise the supplies of indiarubber of the Amazon and of the Orinoco will be worked out through the increasing demand.

It says a great deal for the precautionary sanitary regulations in our convict prisons, when we can go right through last year's report without finding any notification of mortality from erysipelas, small pox, or other eruptive fevers. Indeed, when one remembers that the daily average of persons in penal servitude amounts to something like 3,832, this must be looked upon as a matter of congratulation to the authorities. Neither in this report do we find any case of suicide entered, nor any record of sudden deaths. Many of the prisoners when they are first admitted are found to be suffering from heart disease, but the regular life, the freedom from care and anxiety have been proved in many instances to be conducive of an improvement in the convict's health, as time goes on, whilst their labour is all meted out with due consideration and regard to their physical capacities. "The worst," says Dr. Smalley, of Parkenhurst, "has happened that could happen to them; they are free from suspense and worry, and they lead a placid, regular, and to all appearance, a contented life," and much other testimony to the same effect we find within the pages of this report which has recently been issued.

Although we find that opinions are readily forthcoming on the subject of the physiological effects of tobacco smoke, yet actual statistics on this much controverted subject assume considerably lesser proportions. Dr. Jay Seaver has recently contributed some interesting facts concerning the effect which tobacco produces in the physical development of smokers; facts which are based on a series of observations made on a class of 187 college men during their first and final year. The growth of the men in four of the principal anthropometrical items, of varied character, is as follows:—

| | Weight. Pounds. | Height. Inches. | Chest girth. Inches. | Lung cpcity. Cubic inches. |
|---|---|---|---|---|
| Non-users ...... | 11·87 ... | ·894 ... | 1· 74 ... | 21· 6 |
| Irregular users | 11·06 ... | ·788 ... | 1· 43 ... | 14·45 |
| Habitual users | 10·66 ... | ·721 ... | 1·276 ... | 12·16 |

If this growth be expressed in the form of percentage it will be seen that in weight the non-user increased 10·4 per cent. more than the regular user, and 6·6 per cent. more than the occasional user. In the growth of height the non-user increased 24 per cent. more than the user, and 14 per cent. more than the occasional user. In the growth of chest girth the non-user has an advantage over the regular user of 26·7 per cent., and over the occasional user of 22 per cent., but in capacity of lungs the growth is in favour of the non-user by 77·5 per cent. when compared with the regular user and 49·5 per cent. when compared with the irregular user. Dr. Jay Seaver, the author of these observations, is of opinion that whilst the use of tobacco has already been recognised as injurious to the respiratory track, the element of its influence in checking growth in this and in other directions has not been sufficiently recognised.

